# Book Reviews in Brief

**Published:** 1913-04-05

**Authors:** 


### Book Reviews in Brief.

Life of Sir William Tennant Gairdner. By George Alexander Gibson, M.D. (Glasgow: James Maclehose and Sons, Publishers to the University. 1912.)

Dr. Gibson has undertaken a labour of love in compiling this Life of the late Sir William Gairdner, and old Glasgow men who remember the personality and teaching of the late Regius Professor of Medicine will thank him for having so successfully accomplished his work. But to the general medical reader the book hardly appeals. Gairdner was not a leader in the profession in the sense that Lister, Gull, and Bright were leaders, and, although his personal influence was undoubtedly felt by many medical men and by successive terms of students, there is little in Dr. Gibson's book that justifies the conclusion that he was a genius. After making allowance for the natural enthusiasm of the friend and sympathetic biographer, the impartial reader is led to believe that Gairdner was a conscientious teacher, a hard-working consultant, and a good observer. The book contains excellent portraits and reprints of some of Gairdner's papers, but its most interesting pârt is not the scientific disquisitions but the personal touches. To Glasgow men, at least, Dr. Gibson's life of the old master will be very welcome, and will be whole-heartedly appreciated.

Essays and Clinical Studies. By F. G. Crookshank, M.D. (London: H. K. Lewis. 1912. Pp. 245. Price 7s. 6d.)

Flatulence and Shock. By F. G. Crookshank, M.D. (London: H. K. Lewis. 1913. Pp. 47. Price 2s.)

Essayists like Dr. Crookshank are too rare at the present day. Whatever criticisms may be passed on his theories and deductions, these essays possess in a high degree that elusive quality of readableness. The author has a crispness of style and a happy faculty of keeping the reader wide awake that make his literary excursions not only a refreshing but a lasting pleasure. It has been Dr. Crookshank's good fortune to have held public health appointments and yet to have remained an observant pupil in the school of general practice. Thus his essays cover a wide field, and his subjects are approached in a delightfully broad-minded spirit. The first volume is made up of a collection of previously published papers covering such diverse subjects as Ramazzini, insanity, and the infectious fevers. Of the essays on scarlet fever and diphtheria we may say that they are worthy of careful study by all general practitioners. Two recent papers have now been issued in a separate volume, which is full of practical and helpful suggestions on these two commonplace but difficult subjects.

The Milk Question. By M. J. Rosenau, Professor of Preventive Medicine and Hygiene, Harvard Medical School. (London: Constable and Co. 1912. Pp. 309. Price 7s. 6d. net.)

This book is one which every elector, parliamentary and municipal, ought to read, as well as every parent. It contains not merely dogmatic assertions on the enormously varied aspects of the pure milk problem, but a carefully reasoned exposition of them as well, couched in language which any intelligent and interested layman can understand. The mingling of common sense and scientific precision is a feature which is especially valuable; for although these two qualities ought always to go together, they do not always do so. If only every member of Parliament and every borough councillor could be forced to master the contents of this book there might be a chance of the country obtaining legislation upon which all responsible opinion could agree, vastly to the profit of the community; but this, it seems, is asking too much.

